# Modified Alvarado Scoring System as a diagnostic tool for Acute Appendicitis at Bugando Medical Centre, Mwanza, Tanzania

**DOI:** 10.1186/1471-2482-11-4

**Published:** 2011-02-17

**Authors:** Emmanuel S Kanumba, Joseph B Mabula, Peter Rambau, Phillipo L Chalya

**Affiliations:** 1 Department of Surgery, Weill-Bugando University Collages of Health Sciences, P.O. Box 1464, Mwanza, Tanzania; 2 Department of Pathology, Weill-Bugando University Collages of Health Sciences, P.O. Box 1464, Mwanza, Tanzania

## Abstract

**Background:**

Decision-making in patients with acute appendicitis poses a diagnostic challenge worldwide, despite much advancement in abdominal surgery. The Modified Alvarado Scoring System (MASS) has been reported to be a cheap and quick diagnostic tool in patients with acute appendicitis. However, differences in diagnostic accuracy have been observed if the scores were applied to various populations and clinical settings. The purpose of this study was to evaluate the diagnostic value of Modified Alvarado Scoring System in patients with acute appendicitis in our setting.

**Methods:**

A cross-sectional study involving all patients suspected to have acute appendicitis at Bugando Medical Centre over a six-month period between November 2008 and April 2009 was conducted. All patients who met the inclusion criteria were consecutively enrolled in the study. They were evaluated on admission using the MASS to determine whether they had acute appendicitis or not. All patients underwent appendicectomy according to the hospital protocol. The decision to operate was the prerogative of the surgeon or surgical resident based on overall clinical judgment and not the MASS. The diagnosis was confirmed by histopathological examination. Data was collected using a pre-tested coded questionnaire and analyzed using SPSS statistical computer software.

**Results:**

A total number of 127 patients were studied. Their ages ranged from eight to 76 years (mean 29.64 ± 12.97). There were 37 (29.1%) males and 90 (70.9%) females (M: F = 1:2.4). All patients in this study underwent appendicectomy. The perforation rate was 9.4%. Histopathological examination confirmed appendicitis in 85 patients (66.9%) and the remaining 42 patients had normal appendix giving a negative appendicectomy rate of 33.1% (26.8% for males and 38.3% for females). The sensitivity and specificity of MASS in this study were 94.1% (males 95.8% and females 88.3%) and 90.4% (males 92.9% and females 89.7%) respectively. The Positive Predictive Value and Negative Predictive Value were 95.2% (males 95.5% and females 90.6%) and 88.4% (males 89.3% and females 80.1%) respectively. The accuracy of MASS was 92.9% (males 91.5% and females 87.6%).

**Conclusion:**

The study shows that use of MASS in patients suspected to have acute appendicitis provides a high degree of diagnostic accuracy and can be employed at Bugando Medical Centre to improve the diagnostic accuracy of acute appendicitis and subsequently reduces negative appendicectomy and complication rates. However, additional investigations may be required to confirm the diagnosis in case of atypical presentation.

## Background

Acute appendicitis is one of the most common causes of abdominal surgical emergencies with a lifetime prevalence of approximately 1 in 7 worldwide [1]. It is associated with high morbidity and occasionally morbidity related to failure of making an early diagnosis. It has been estimated that approximately 6% of the population will suffer from acute appendicitis during their lifetime; therefore, much effort has been directed toward early diagnosis and intervention [2,3].

Early diagnosis and prompt operative intervention is the key for successful management of acute appendicitis. However, the picture of acute appendicitis may not be classical, and in such situations, a policy of early intervention to avoid perforation may lead to high negative appendicectomy rates [4,5]. Difficulties in diagnosis arise in very young, elderly patients and females of reproductive age because they are more likely to have an atypical presentation, and many other conditions may mimic acute appendicitis in these patients [6]. In such cases, clinical examination should be complemented with laparoscopy or diagnostic imaging such as Ultrasound scan or CT scan to exclude diseases other than appendicitis.

A negative appendicectomy rate of 20-40% has been reported in literature and many surgeons advocate early surgical intervention for the treatment of acute appendicitis to avoid perforation, accepting a negative appendicectomy rate of about 15-20% [7].

Removing normal appendix is an economic burden on both patients and health resources. Misdiagnosis and delay in surgery can lead to complications like perforation and finally peritonitis [8]^.^

Many scoring systems for the diagnosis of acute appendicitis have been tried, but most of these are complex and not feasible in emergency setting [9]. The MASS has been shown by recent studies to be easy, simple and cheap diagnostic tool for supporting the diagnosis of acute appendicitis especially for junior surgeons [9,10]. However, its application and usefulness in the diagnosis of acute appendicitis has not been evaluated at Bugando Medical Centre; as a result, the rate of negative appendicectomy is not known. The aim of this study is to assess the diagnostic value of MASS in patients with acute appendicitis at Bugando Medical Centre.

## Methods

This was a cross sectional study to evaluate the diagnostic value of MASS in patients presenting with acute appendicitis at the A & E department of Bugando Medical Centre over a period of six months from November 2008 to April 2009. All patients with a clinical diagnosis of acute appendicitis and undergoing appendicectomy during the study period were, after informed consent, consecutively enrolled into the study. Patients with a mass in the right iliac fossa and those who fail to provide information and had no relatives nearby were excluded from the study. Patients who had no histopathological results were also excluded from the study.

Ethical approval to conduct the study was obtained from the WBUCHS/BMC joint institutional ethic review committee before the commencement of the study.

All patients included in the study were initially seen by the admitting registrar or resident surgical student who made the decision to operate. The Principal Investigator scored all the patients according to the variables of MASS (Table 1) and then divided them into two groups. Group I included patients with MASS of seven and above (patients likely to have acute appendicitis) and Group II were patients with MASS below seven (patients unlikely to have acute appendicitis). The Principal Investigator did not influence the management of the patient and the decision to operate was not based on MASS but the clinical impression by the clinician taking charge of the patient. Abdominal ultrasound was performed in case of atypical presentation. All patients underwent emergency appendicectomy and all appendices removed at operation were sent for histopathology. The diagnosis of acute appendicitis was confirmed by histopathological examination. Data was collected using a coded, pre-tested questionnaire and analyzed using SPSS statistical software version 11.5. The MASS groups were cross-tabulated against histology, the gold standard. Then, the sensitivity, specificity, accuracy, Positive Predictive Value (PPV) and Negative Predictive Value (NPV) and accuracy were determined in males and females.

**Table 1:** Modified Alvarado Scoring System (MASS) F

Symptoms	Score
Migratory right iliac fossa pain	1
Nausea/Vomiting	1
Anorexia	1

**Signs**	

Tenderness in right iliac fossa	2
Rebound tenderness in right iliac fossa	1
Elevated temperature	1

**Laboratory findings**	

Leucocytosis	2
**Total**	**9**

## Results

A total of 127 patients were enrolled in the study. Their ages ranged from eight to 76 years (mean 29.64 ± 12.97). There were 37 (29.1%) males and 90 (70.9%) females (M: F = 1:2.4). The duration of illness of the study population ranged from 1 day to 42 days with a mean of 10.68 days and standard deviation of 8.46 days. The median was 7 days and the mode was 4 days. There was a significant association between the duration of illness and perforation rate [Odds Ratio = 8.442, 95% C.I. (1.625-43.981), p-value = 0.003]. The MASS of the study population ranged from 3 to 9. (Mean 6.78 ±1.51). The median and the mode were 7.00 and 8.00 respectively. In this study, 84 patients (66.1%) had a MASS of seven and above and the remaining 43 patients (33.9%) had MASS below seven. All patients in this study underwent appendectomy. Of these, inflamed appendix was the most common operative findings affecting 80 patients (62.9%). Twelve patients (9.4%) had perforated appendices, six patients (4.7%) had gangrenous appendices and four patients (3.1%) had appendicular abscess. None of these appendicular complications was missed by MASS.

Other operative findings in the study occurred in 14 patients (11.0%) (Table 2).

**Table 2:** Operative findings

Operative findings	Frequency	Percentage
Inflamed appendix	80	62.9
Gangrenous appendix	6	4.7
Perforated appendix	12	9.4
Appendicular abscess	4	3.1
Normal appendix	11	8.7
Other findings	14	11.0
Total	127	100

Histological examination confirmed appendicitis in 85 patients (66.9%). The remaining 42 patients were found to have normal appendix giving a negative appendicectomy rate of 33.1% being 26.8% and 38.3% for males and females respectively. Other histological findings included carcinoid tumor in one patient (25%), S. haematobium in one patient (25%), mucocele of the appendix in one patient (25%) and lymphoid hyperplasia in one patient (25%) and all were reported as chronic specific appendicitis (Table 3).

**Table 3:** Histological findings

Histological findings	Frequency	Percentage
Normal appendix	42	33.1
Acute appendicitis	40	31.5
Suppurative appendicitis	15	11.8
Chronic non specific appendicitis	26	20.5
Others (chronic specific appendicitis)	4	3.1
Total	127	100

The sensitivity and specificity of MASS in this study was 94.1% (males 95.8% and females (88.3%) and 90.4% (males 92.9% and females 89.7%) respectively. The PPV was 95.2% (males 95.5% and females 90.6%) and NPV was 88.4% (males 89.3% and females 80.1%. The accuracy of MASS was 92.9% (males 91.5% and females 87.6%) (Table 4).

**Table 4:** MASS versus histological findings

MASS	Histological findings		Total
		
	Appendicitis	No appendicitis	
≥ 7	80	4	84
< 7	5	38	43
Total	85	42	127

MASS showed high sensitivity (95.8%) and specificity (94.1%) in adult (16-60 years) than in children (93.3%/93.3%) and geriatric (85.7%/80.0%) age groups (Table 5)

**Table 5:** MASS versus histological findings in different age groups

MASS/age (in years)	Appendicitis			No appendicitis			Total
		
	0-15	16-60	> 60	0-15	16-60	> 60	
**≥ **7	28(93.3%)	46 (95.8%)	6 (85.7%)	1 (6.7%)	1(20.0%	2(20.0%)	84
< 7	2 (6.7%)	2 (4.2%)	1 (14.3%)	14(93.3%)	16(94.1%	8(80.0%)	43
Total	30 (100%)	48(100%)	7 (100%)	15(100%)	17(100%	10(100%)	127

Simple appendicitis was more common in all age groups, whereas children aged (0-15) had significant higher perforation rate compared to other age groups (P = 0.0021). Table 6

**Table 6:** Simple versus complex appendicitis in different age groups

Age group (in years)	**Simple appendicitis (non-perforated**)	Complex appendicitis (perforated)	Total
0-15 (Children)	17 (63.0%)	10 (27.0%)	27 (100%)

16-60 (Adult)	47 (92.2%)	4 (7.8%)	51 (100%)

> 60 (Geriatric)	5 (71.4%)	2 (28.6%)	7 (100%)

Total	69	16	85

## Discussion

The use of MASS in the diagnosis of acute appendicitis has been reported to improve the diagnostic accuracy and consequently reduces negative appendicectomy and complication rates [9,10]. This study was conducted to evaluate the diagnostic value of Modified Alvarado Scoring System in patients with acute appendicitis in our setting.

The age distribution in our study was similar to other studies [9-11]. The female preponderance in this study is in agreement with other studies [11,12]. Studies in Kenya, Nigeria and Ethiopia found a male dominance [13-15]. The reason for the difference in sex distribution in these studies may be attributed to the fact that female patients with right iliac fossa pain have a wide range of differential diagnoses as a result acute appendicitis may be over-diagnosed in this gender group. In this case, therefore, additional investigations may be required in female patients to confirm the diagnosis of acute appendicitis.

In this study, the duration of illness in majority of patients was four days and majority of patients reported to the hospital and seen by the admitting doctor in more than 24 hours after the onset of illness. This observation concurs with other reports [11,12]. The reasons for delay in seeking medical consultation in this study may be attributed to delay in referral from peripheral hospitals, lack of money to pay for the medical services and for transport. Delayed presentation may also be due to misdiagnosis or fear of surgery as a result they are treated conservatively with analgesics and antibiotics to mask the symptoms. Delayed presentation is associated with increased morbidity and mortality due to appendiceal perforations and peritonitis.

The rate of perforation in our study was 9.4%, which is comparable to other reported rates [16,17]. However, much higher perforation rates have been reported from other centres in Nigeria [18]. In developing countries, rates of between 6-65% have been quoted [19]. Delayed presentation, fulminate disease, misdiagnosis, or failure to accept surgical treatment, are contributory factors to high perforation rates. Perforation rates are much higher in the very young and the elderly, where diagnosis is often difficult leading to perforation rates as much as 80% in some reported series [20,21]. In our study, the perforation of appendices occurred mostly in patients with MASS ≥ seven and in the children aged 0-15 years. Therefore, a more aggressive approach should be used in patients with high scores and in advanced age individuals and children.

The overall negative appendicectomy rate (33.1%) in our study was found to be higher than that reported in Nigeria [22,23]. The reason for high negative appendicectomy rate in our series may be due to appendicectomies that were done to patients who presented with other conditions mimicking acute appendicitis. Our figures for negative appendicectomy rate in the present study were found to be slightly higher in females (38.3%) than in males (26.8%). This is because misdiagnosis may have occurred in females of reproductive age group where other pelvic diseases could make diagnosis difficult. In such cases, MASS should be complemented with diagnostic procedure like laparoscopy or imaging such as Ultrasound scan or CT scan to minimize the rate of negative appendectomy [6]. However, a large population based study suggested that the rate of negative appendicectomy (15-20%) has not declined for 15 years despite the increasing use of such tests [24].

The histopathological findings in our study were not different from other reports in developing countries. However, the finding of appendiceal schistosomiasis due to Schistosoma haematobium, in contrast to *Schistosoma mansoni *as reported in some series is surprising. Similar histological finding was also reported by others [19,25]. This could be explained by high endemicity of *S. haematobium *in Mwanza region which is along the shore of Lake Victoria and therefore the chance of *S. haematobium *infestation is high. *S. haematobium *usually affects the bladder, prostate, rectum, and the cervix but in endemic areas, it can be found in the appendix inducing chronic inflammation that could manifest as appendicitis [26].

The present study has shown that MASS provides high degree of sensitivity, specificity, PPV, NPV and accuracy in the diagnosis of acute appendicitis, which is in agreement with findings reported by others [7,27], but in sharp contrast to what was observed in Kenya [13].

Our study also revealed that MASS is more helpful in male patients by showing lower negative appendicectomy rate and high positive predictive value for male patients as compared to females. In females, additional investigations may be required to confirm the diagnosis. Literatures also support this observation [28-30].

One limitation of our study is that HIV infection which has great effect on the WBC count was not tested in our patients, and this could have affected the results of the study.

## Conclusion and Recommendations

The present study has shown that MASS provides high degree of sensitivity, specificity, PPV, NPV and accuracy in the diagnosis of acute appendicitis and has found to be more helpful in male patients by showing lower negative appendicectomy rate and high positive predictive value for male patients as compared to females. It is therefore recommended that:-

• MASS should be used at BMC to improve the diagnostic accuracy of acute appendicitis and subsequently reduce negative appendicectomy and complication rates.

• The use of MASS in the diagnosis of acute appendicitis in female patients should be supplemented by additional investigations like abdominal ultra sound or laparoscopy

• A MASS score above 7 should indicate appendectomy without the need for further imaging

## Competing interests

The authors declare that they have no competing interests.

## Authors' contributions

ESK - Study design, data analysis, manuscript writing & editing, JBM - Data analysis, manuscript writing & editing, PR - Data analysis & manuscript writing & editing and PLC - Study design, data analysis, manuscript writing & editing. All the authors read and approved the final manuscript.

## Pre-publication history

The pre-publication history for this paper can be accessed here:

http://www.biomedcentral.com/1471-2482/11/4/prepub
